# Determination of immunogenic proteins in biopharmaceuticals by UHPLC–MS amino acid analysis

**DOI:** 10.1186/s13065-019-0581-z

**Published:** 2019-05-10

**Authors:** Juraj Piestansky, Jaroslav Galba, Dominika Olesova, Branislav Kovacech, Andrej Kovac

**Affiliations:** 1 Department of Pharmaceutical Analysis and Nuclear Pharmacy, Faculty of Pharmacy, Comenius University in Bratislava, Odbojarov 10, 832 32 Bratislava, Slovak Republic; 2 Toxicological and Antidoping Center, Faculty of Pharmacy, Comenius University in Bratislava, Odbojárov 10, 832 32 Bratislava, Slovak Republic; 3 AXON Neuroscience R&D, Dvorakovo Nabrezie 10, 845 10 Bratislava, Slovak Republic; 4 Institute of Neuroimmunology, Slovak Academy of Science, Dubravska Cesta 9, 845 10 Bratislava, Slovak Republic

**Keywords:** Amino acids, Ultra high-performance chromatography, Mass spectrometry, Derivatization, Biopharmaceuticals, Keyhole limpet hemocyanin, Etanercept, Quality control

## Abstract

Nowadays, there is a growing interest in innovative and more efficient therapeutics—biopharmaceuticals, based on peptides or proteins. There are increased demands on quality control of such therapeutics. One of the methods usually used for characterization and quantification of biopharmaceuticals is amino acid analysis. In this work, a modern advanced analytical method based on precolumn derivatization and reversed-phase ultra high-performance liquid chromatography in combination with single quadrupole mass spectrometer was developed for amino acid analysis in different protein samples—model sample of bovine serum albumin, sample of strong immunogenic protein keyhole limpet hemocyanin, and sample of drug etanercept present in commercially available biopharmaceutical Enbrel. The method used isotopically labeled internal standards and was validated according to the International Council for Harmonisation guideline. The developed method was characterized by favorable performance and validation parameters, such as time of analysis (6 min), specificity, linearity (r^2^ ≥ 0.99), limit of detection (0.009–0.822 µM), limit of quantification (1–2.5 µM), accuracy (recovery in the range 90–102.8%), intra-day (RSD in the range 0.25–11.97%) and inter-day precision (RSD in the range 1.67–11.57%), or stability (RE ≤ 12%). According to these findings, the developed amino acid analysis approach is suitable for routine use in areas of peptide/protein quantification, such as quality control laboratories of biopharmaceutical companies.
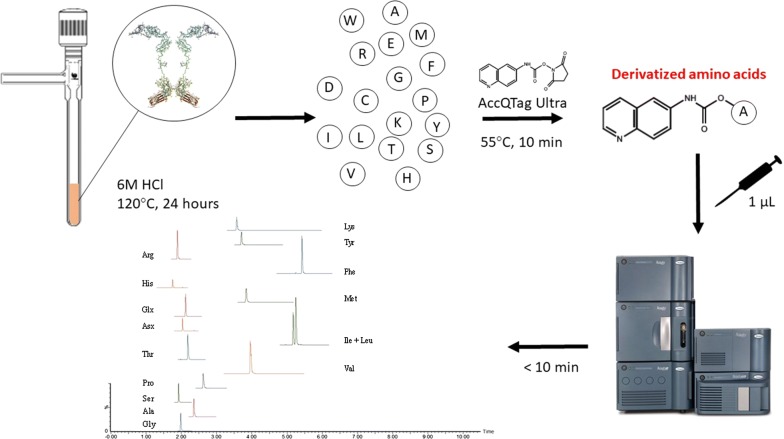

## Introduction

**Fig. 1:**
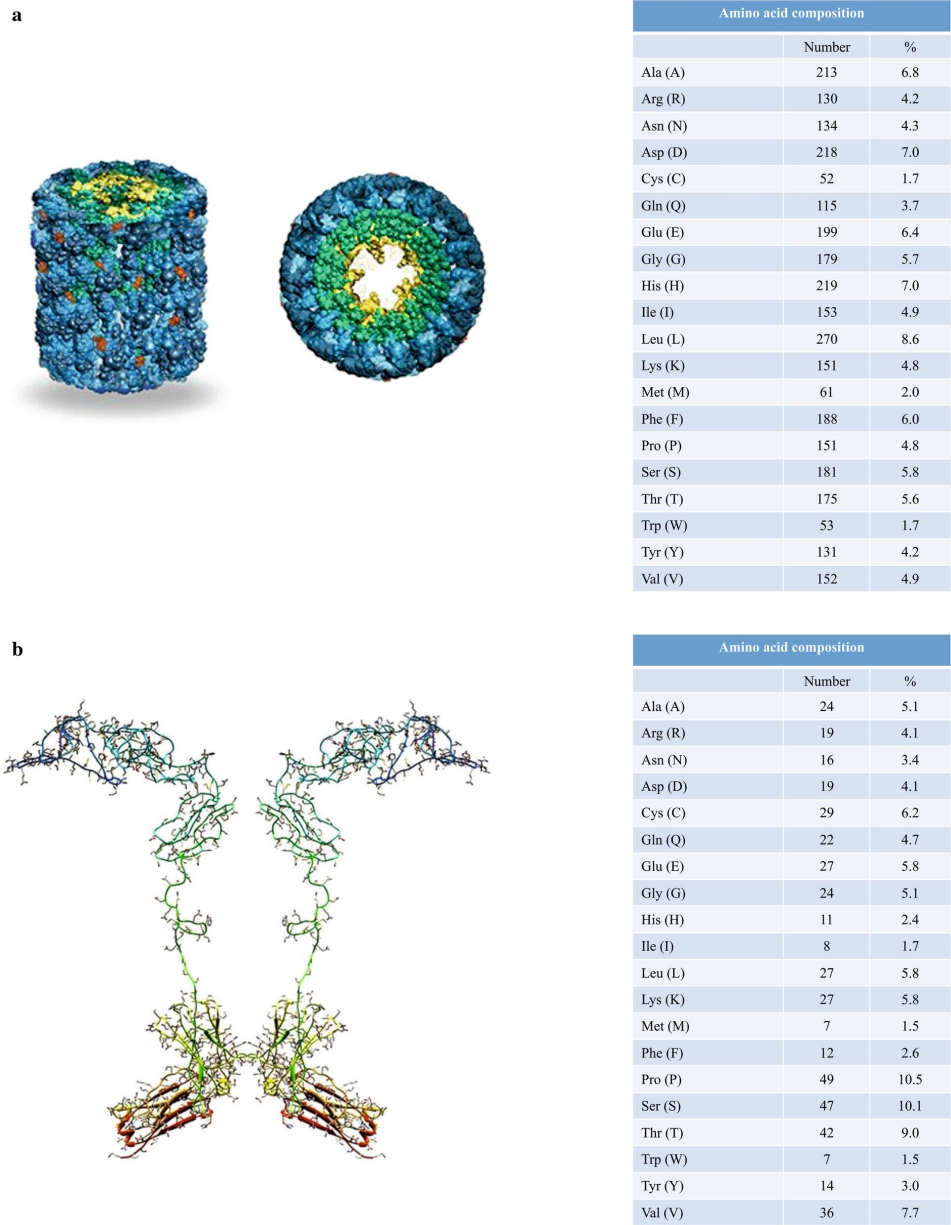
Illustrative scheme of the keyhole limpet hemocyanin—KLH (**a**) and the biopharmaceutical drug etanercept (**b**) with the corresponding abundance of each amino acid (adapted from [3, 4])

The use of novel biologics, peptide therapeutics, therapeutic peptide conjugates, and therapeutic proteins continues to increase. There is a growing tendency to use them in treatment of several diseases such as various types of cancer, inflammation or neurodegeneration. Keyhole limpet hemocyanin (KLH) is an extracellular respiratory protein which is isolated from the Californian giant keyhole limpet Megathura crenulata. KLH protein consists of two structurally and physiologically distinct isoforms—KLH1 and KLH2, each based on a subunit with a molecular weight of approximately 400 kDa [1, 2]. The amino acid sequence and illustrative configuration of the protein are in detail described in Fig. 1a. This protein acts as a potent immune activator, and therefore it is widely used as hapten-carrier and immune stimulant in research and clinical studies. Recently, there are 67 clinical studies directly associated with KLH in immunotherapy of different cancer diseases (e.g. bladder cancer, lung cancer, myeloma, prostate cancer, leukemia, breast cancer, etc.), multiple sclerosis, autoimmune disorders, HIV infections, neurodegenerative diseases (such as Alzheimer’s disease, Parkinson´s disease) or opioid use disorder [3]. In many experimental, pre-clinical or clinical studies the active substance (chemical drug or peptide) are chemically conjugated to the KLH that represents a carrier protein [4–6]. The knowledge of the exact concentration of carrier proteins and conjugated peptide in such vaccines is critical not only from regulatory but also from a clinical point of view. The information about the concentration of therapeutic peptide/protein can be used to adjust the effective and safe dose. On the other side, the information about the concentration of the carrier protein is necessary for quality assurance of pharmaceutical ingredients.

Biopharmaceutical Enbrel contains drug etanercept—the dimeric fusion protein consisting of the extracellular ligand-binding portion of the human 75 kDa (p75) tumor necrosis factor receptor (TNFR) linked to the Fc portion of human IgG1. Etanercept is produced by recombinant DNA technology in a Chinese hamster ovary (CHO) mammalian cell expression system and consists of 934 amino acids. The amino acid sequence of monomer part of etanercept is in detail described in Fig. 1b. The first FDA approval for Enbrel was in 1998 for the treatment of moderate to severe rheumatoid arthritis. Moreover, between the years 1999 and 2004 Enbrel was also approved for the treatment of moderate to severe polyarticular juvenile rheumatoid arthritis, psoriatic arthritis, ankylosing spondylitis and moderate to severe plaque psoriasis. The mechanism of action of etanercept is based on modulation of the proinflammatory cytokine TNF-α activity by competitively binding to both cell-surface and soluble TNF-α, preventing the interaction of TNF-α with endogenous receptors located on the cell surface [7].

In general, biopharmaceuticals are very effective therapeutics characterized by higher potency and reduced side effects. Although the safety of biopharmaceuticals is on high level, they can elicit an immune response, which can lead to generalized immune effects such as allergy or anaphylaxis. These and also other side effects very often depend on the applied dose of the therapeutics. The quality control of such biopharmaceuticals is therefore a crucial step and development of simple and reliable analytical methods for quantitative analysis of therapeutic peptides or proteins are demanded.

Amino acid analysis (AAA) represents an essential tool in the field of foodomics, proteomics, metabolomics and also in the growing world market with biopharmaceuticals. In general, AAA can be used to quantify proteins and peptides, to determine the identity of proteins or peptides based on their amino acids composition, to support protein and peptide structure analysis, to evaluate fragmentation strategies for peptide mapping and to detect atypical amino acids that may be present in a protein or peptide. Thanks to this method the characterization of biopharmaceuticals and therapeutic peptides and proteins can be markedly simplified.

The procedure of AAA is based on the hydrolysis of a protein or peptide into its components—free amino acids, and their subsequent chromatographic or electromigration separation. Hydrolysis is performed under acidic conditions (usually 6 M HCl) at elevated temperature (110–165 °C) typically for 1 to 24 h. The demands on time and temperature conditions of the hydrolysis procedure are very often affected by the nature of the desired analysis and the primary sequence of the analyzed protein/peptide. The main difficulties concerning acid hydrolysis are associated with the cleavage of some of the amide bonds between aliphatic amino acids. The resistance of the Ala–Ala, Ile–Ile, Val–Val, Val–Ile, Ile–Val, and Ala–Val linkages to the suitable hydrolysis conditions was observed [8]. This discrepancy can be solved by extending the time of hydrolysis procedure.

Various separation methods have been developed for the analysis of amino acids in different matrices (e.g., liquid chromatography—LC, gas chromatography—GC, capillary electrophoresis—CE), and impressive achievements have been made in the field of separation and detection of amino acids. Among the separation methods, liquid chromatography prevailed in the AAA [9]. Many different chromatographic techniques have been used to separate and quantify amino acids. In general, recent chromatographic approaches in AAA are based on three major strategies: (a) direct analysis of free amino acids, (b) analysis which combines ion-exchange chromatography separation with post-column derivatization and (c) strategies based on pre-column derivatization. The simplest way how to perform the AAA is direct analysis. In this case, the amino acids are mainly separated by ion-exchange chromatography (IEC) [10], ion-interaction chromatography (IIC) [11] or hydrophilic interaction chromatography (HILIC) [12, 13]. The main challenge in such type of analysis is lack of a moiety in the chemical structure of these molecules which allows UV, fluorescence and electrochemical detection.

Moreover, the amphoteric nature of amino acids very often results in poor retention in case of reversed-phase (RP) liquid chromatography (LC) methods. Suitable strategies to overcome these disadvantages are based on derivatization procedures performed before the analysis and on the use of highly selective, specific and sensitive detection by mass spectrometry (MS). Some interesting original or excellent review papers were recently published which describe the benefits of pre- or post-column derivatization strategies of amino acid analysis [14–16]. Moreover, in some cases, the intrinsic fluorescence of aromatic amino acids was used for quantification of hydrolyzed peptides and proteins [17].

It was demonstrated, that the LC–MS approach offers very good efficiency, ease of use and high speed of analysis [18]. Moreover, MS has recently become the most common detection method in bioanalysis. According to these facts, the precolumn derivatization strategies in combination with LC–MS analysis are often preferred in the analysis of AAA.

Nowadays, there are several different reagents and reagent kits available for derivatization of amino acids to facilitate and improve LC–MS analysis. Many of them are predominantly used in the field of bioanalysis and clinical analysis—e.g. O-phthalaldehyde (OPA) [19], aTRAQ reagents [20–22], iTRAQ reagents [23], chloroformate [24], n-propyl chloroformate [25], bromobutane [26], 9-fluorenylmethyl chloroformate (FMOC-Cl) [27], 5-(dimethylamino)-naphthalene-1-sulfonyl chloride (Dansyl-Cl) [28], dimethyl-1,1,1-d_3_-aminobutyryl succinimide hydrochloride (d_3_-DMABS), and dimethyl-d_6_-aminobutyryl succinimide hydrochloride (d_6_-DMABS) [29], phenylisothiocyanate (PITC) [30], 3-aminopyridyl-N-hydroxysuccinimidyl carbamate [31], 6-aminoquinolyl-*N*-hydroxysuccinimidyl carbamate (AQC) [32–34], or 5-aminoisoquinolyl-*N*-hydroxysuccinimidyl carbamate (5-AIQC) [35]. It is because of demand for very effective, sensitive and highly reliable analytical methods in such areas.

**Fig. 2:**
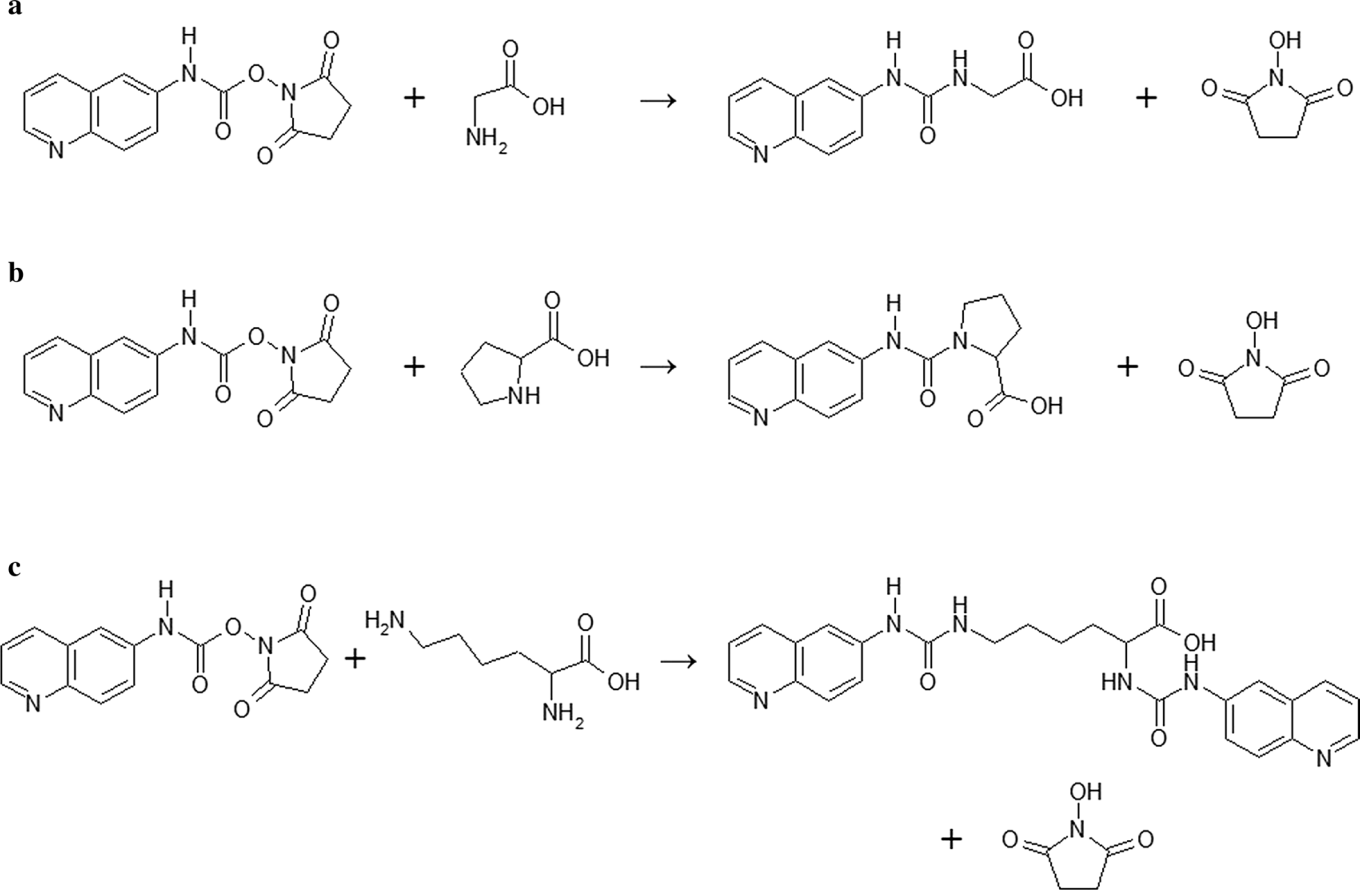
Scheme of derivatization procedure of three different amino acids—glycine (**a**), proline (**b**) and lysine (**c**) with the use of Acc.QTag Ultra derivatization reagent and generation of appropriate products of the reaction

Derivatization of amino acids performed with the use of 6-aminoquinolyl-*N*-hydroxysuccinimidyl carbamate, commercially known as AccQTag Ultra technology, is an accepted procedure that enables fast (the reaction is completed within 10 min) transformation of primary and secondary amines into highly stable fluorescent derivatives [36, 37]. During the derivatization procedure AQC reacts with primary and secondary amines resulting in the formation of a fluorescent urea derivative with high stability. The excess reagent is then rapidly hydrolyzed into 6-aminoquinoline, *N*-hydroxysuccinimide, and carbon dioxide. The reaction mechanism of the derivatization procedure is in details illustrated in Fig. 2.

Here we propose a fast, simple, sensitive, and selective targeted AAA based on ultra high-performance liquid chromatography (UHPLC–MS) assay for quantitative analysis of peptides and total protein content in samples with biopharmaceutical potential. To the best of our knowledge, up to now, there have been no studies dedicated to the use of AccQTag Ultra derivatization in connection with UHPLC coupled to simple single quadrupole mass spectrometry detector (QDa) for fast amino acids identification and quantification for biopharmaceutical purposes. Such an approach is relatively easy, cheap, time-saving and there is no requirement for highly trained personnel. Therefore, it represents a very attractive approach for quality control and clinical laboratories.

## Materials and methods

### Instrumentation and analytical conditions

The chromatographic apparatus consisted of an ACQUITY UPLC H-Class chromatographic system with a quaternary gradient pump, autosampler, and column thermostat. The chromatographic system was on-line coupled with mass single-quadrupole detector (QDa) with an electrospray ionization source (Waters, Prague, Czech Republic). Data were acquired, calibrated and quantified by MassLynx software (Waters). Chromatographic separation was performed on Cortecs UPLC C18 column (2.1 mm × 100 mm, 1.6 µm particle size). The column temperature was set for 55 °C. Mobile phase A consisted of 0.1% formic acid (FA) in LC–MS water and mobile phase B was 0.1% formic acid (FA) in acetonitrile. The gradient program was as follows: 1% B (0–0.7 min), increased to 13% B (0.7–1.3 min), to 15% B (1.3–3.7 min), to 40% B (3.7–7.0 min), to 95% B (7.0–8.0), hold on 95% B (8.0–9.0 min), returning to 1% B (9.0–9.8 min) and re-equilibrating (9.8–10.5 min). Total run time was 10.5 min. The flow rate was 0.5 mL min^−1^, and the injection volume was 1 µL. The temperature of the autosampler was set at 10 °C throughout the analysis.

Positive electrospray ionization mode (ESI+) was used for the MS detection of derivatized amino acids, and the appropriate m/z values of ions of each analyte and its deuterated internal standard were monitored in the single ion-monitoring (SIM) mode. The following MS conditions were applied. The capillary voltage was set at 0.8 kV, the source block and probe temperatures were 150 °C and 600 °C, respectively. Nitrogen was used as the desolvation and nebulizer gas. The cone voltage was set to 15 V.

### Chemicals and samples

Amino acid standard that included 17 amino acids (l-alanine, l-arginine, l-aspartic acid, l-cystine, l-glutamic acid, glycine, l-histidine, l-isoleucine, l-leucine, l-lysine, l-methionine, l-phenylalanine, l-proline, l-serine, l-threonine, l-tyrosine, l-valine) and standards of l-asparagine, l-glutamine, and l-tryptophan were obtained from Sigma Aldrich (Steinheim, Germany). Isotopically labeled amino acids for use as internal standards were from Cambridge Isotope Laboratories (Massachusetts, USA). Formic acid (HFo), acetonitrile, and LC–MS grade water were purchased from Sigma Aldrich (Steinheim, Germany), AccQTag Ultra reagent, AccQTag Ultra borate buffer (pH = 8.6) and Amino Acid Standard Hydrolysate were from Waters Corporation (Prague, Czech Republic). Bovine serum albumin (BSA) was obtained from Sigma Aldrich, keyhole limpet hemocyanine (KLH)—preparation Vacmune^®^, was obtained from biosyn Arzneimittel GmbH (Fellbach, Germany). The commercially available biopharmaceutical Enbrel with the content of drug etanercept was obtained from the company Pfizer (Bratislava, Slovakia).

### Procedures for a sample and standard solution preparation

#### Standard solutions and calibration solutions

A stock solution of 20 amino acids at the concentration level of 500 µM (except of Cys, which concentration level was 250 µM) was prepared by dilution of the commercial amino acids standard mixture and dissolution of the appropriate amount of individual standards of glutamine, asparagine, and tryptophan in 0.1 M HCl. Calibration standards were prepared by dilution of stock solution with LC–MS grade water to give concentration in the range 1–500 µM (2.5–250 µM for cysteine, respectively).

The internal standard stock solution was prepared by dilution of commercially available isotopically labeled amino acids mixture and dissolution of isotopically labeled standards of glutamine, asparagine, and tryptophan in 0.1 M HCl. Work solution of internal standard was prepared by appropriate dilution of the prepared internal standard stock solution.

#### Protein sample preparation

Sample of hydrolyzed amino acid standard from Waters was 10-times diluted with LC–MS grade water, and the concentration of each amino acid presented in the sample was 250 µM, except Cys, which concentration was 125 µM. Model sample of bovine serum albumin (BSA) was prepared by dissolution of the exact amount of BSA in LC–MS grade water, and the final concentration of this solution was 5 mg mL^−1^. The KLH sample (preparation Vacmune^®^) at 20 mg mL^−1^ concentration level was used without any preparation. The sample of commercially available biopharmaceutical Enbrel that contents etanercept at 50 mg mL^−1^ concentration level was used without any preparation steps. The samples of BSA, KLH, and Enbrel then proceeded to the hydrolysis step.

#### Hydrolysis of protein samples

A 100 µL of each protein sample (BSA, KLH–Vacmune^®^, Enbrel) was transferred into the hydrolysis tube and 20 µL of 0.1% phenol solution was added. The tube was after that flushed with nitrogen. Then, 250 µL of 6 M HCl solution was added, and the tube was evacuated. The hydrolysis tube was then placed on the heating block for 24 h at 120 °C. After the hydrolysis step and cooling of the sample to the laboratory temperature, 10 µL of the sample was mixed with 90 µL of AccQTag Ultra borate buffer. This solution was gently mixed, and 5 µL of the sample was taken into the derivatization reaction.

#### Derivatization procedure

The derivatization agent was prepared by addition of 1 mL of acetonitrile into the AccQTag Ultra reagent powder, vortex mixing and dissolving by heating at 55 °C for 15 min. After the preparation of the derivatization agent, 70 µL of borate buffer was added into the 1.5 mL Eppendorf tube. Then 5 µL of prepared sample and 5 µL of the internal standard mixture was transferred into the tube, followed by 20 µL of prepared AccQTag derivatizing reagent solution. This solution was properly vortex mixed and heated at 55 °C for 10 min. Samples were then transferred into the vials and analyzed by LC–MS.

## Results and discussion

This study aimed at the development of a fast, selective and sensitive UHPLC–MS method for amino acid analysis in peptide and protein samples applicable into the biopharmaceutical area. The presented method is based on RP-chromatography and precolumn derivatization procedure by AccQTag Ultra reagent. In general, RP-chromatography is not suitable for separation of polar compounds in their native form. Therefore targeted modification of such molecules is required.

### Method development and optimization

#### Hydrolysis conditions

The first and crucial step in quantitative AA analysis is the hydrolysis step. Several different approaches are described in the European Pharmacopoeia [38]. Acid hydrolysis using 6 M hydrochloric acid containing 0.1–1% phenol is the most common procedure used for proteins and peptides hydrolysis preceding amino acid analysis. The method is specific for all amino acids except cysteine and tryptophan and prevents the halogenation of tyrosine [39]. According to these facts, this procedure was selected as appropriate for our purposes.

The hydrolysis procedure was performed according to the recommendation of European Pharmacopoeia [38]. The suitability of the selected conditions was tested according to the recovery test. The sample of keyhole limpet hemocyanin (KLH)—preparation Vacmune^®^, was taken as a testing substance and the hydrolysis conditions—time and temperature were changed. In general, three different times—20, 24 and 28 h, and three different temperatures—110, 120 and 130 °C were tested. Total protein content was determined and compared with the amount declared by the manufacturer. The results are summarized in Table 1. According to these results, it can be stated that the initial chosen hydrolysis conditions (120 °C, 24 h) are appropriate and variation of the conditions (± 10 °C, ± 4 h) have a negligible influence on complete and reproducible hydrolysis process of the protein. In two cases, the recovery value was slightly higher than 100% but in good agreement with the recommendation of international guidelines for validation. This discrepancy could be associated with the drawbacks of the hydrolysis. The main disadvantage of the protein hydrolysis procedure is the difference in recovery for individual amino acids. The recovery of amino acids from the protein sample is very often affected by complete or partial destruction (tryptophan, cysteine, threonine, serine, and methionine), incomplete bond cleavage (isoleucine, valine) or free amino acid contamination (glycine, serine). Determination of the protein content (protein concentration) in the analyzed sample is therefore problematic. In such cases, the quantification of the protein concentration is calculated with the use of information about the content of well-recovered amino acids presented in the sample. Well-recovered amino acids are aspartate-asparagine (Asx), glutamate-glutamine (Glx), alanine, leucine, phenylalanine, lysine, and arginine. This approach was used in current study.

**Table 1:** Effect of different hydrolysis conditions (temperature and time) on recovery of the tested keyhole limpet hemocyanin (KLH) protein sample

	Expected concentration [mg mL^−1^]	Determined concentration [mg mL^−1^]	Recovery [%]
110 °C/20 h	20	19.56	97.8
110 °C/24 h	20	19.64	98.2
110 °C/28 h	20	19.14	95.7
120 °C/20 h	20	20.00	100.0
120 °C/24 h	20	20.12	100.6
120 °C/28 h	20	19.84	99.2
130 °C/20 h	20	19.76	98.8
130 °C/24 h	20	20.30	101.5
130 °C/28 h	20	19.74	98.7

#### Derivatization optimization

**Fig. 3:**
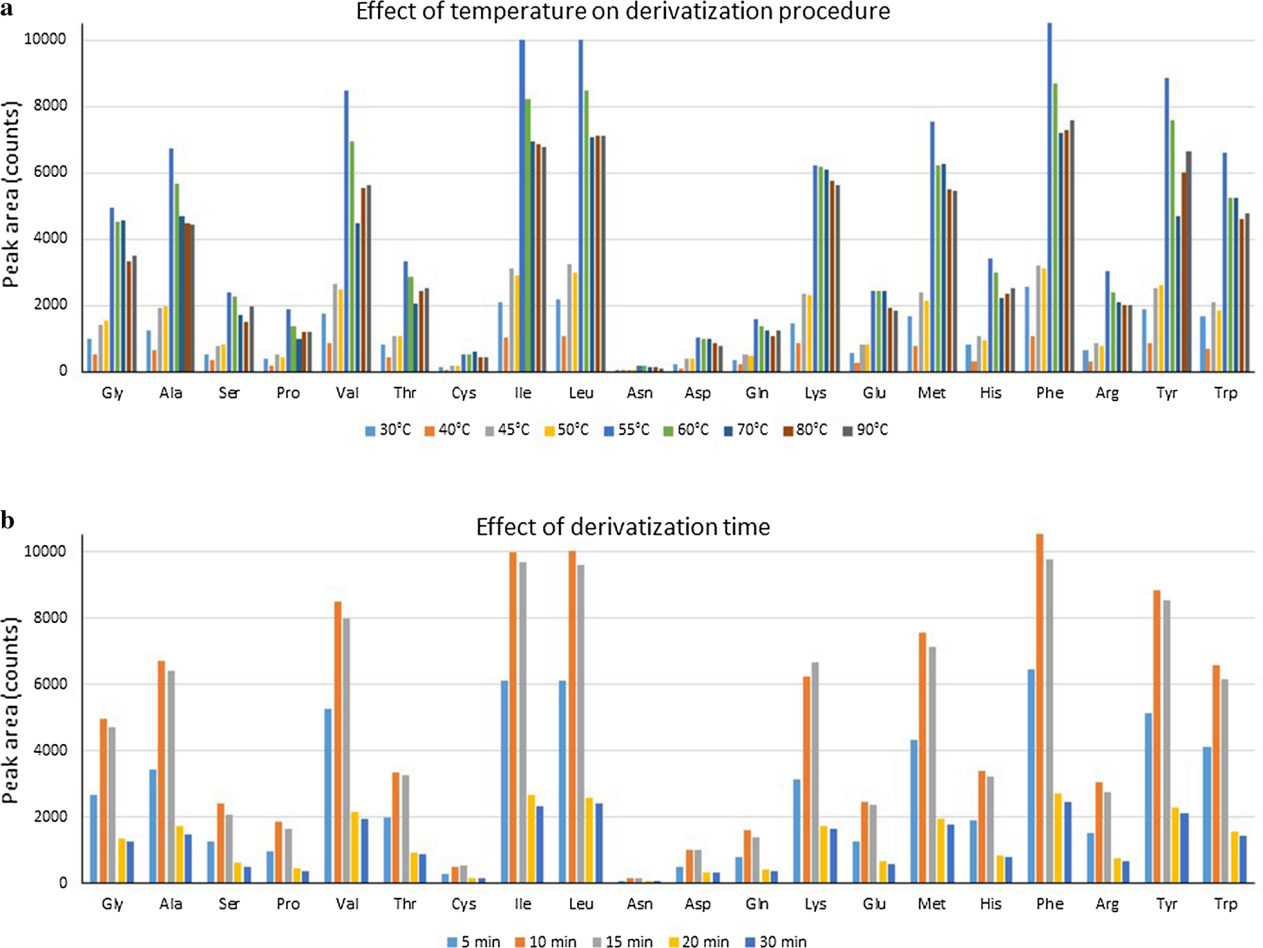
Derivatization reaction optimization

AccQTag Ultra (6-aminoquinolyl-*N*-hydroxysuccinimidyl carbamate) was selected as a derivatization reagent in the amino acid analysis based on the sensitivity, selectivity, simplicity, reaction conditions, efficiency, and commercial availability. The derivatization reaction conditions were optimized to ensure maximum conversion of all amino acids, and therefore, reaction time and reaction temperature of derivatization procedure were studied in more depth. The results from these separate studies are summarized in Fig. 3. At first, the temperature in the range of 30–90 °C was investigated. According to the obtained results, the temperature of 55 °C was selected for the further optimization process. After that, the reaction kinetics at 55 °C was monitored from 5 to 30 min. It was demonstrated, that the derivatization time 10 min is sufficient for completion of the derivatization reaction and there is no interference of excess amount of derivatization reagent with the chromatography and analysis of amino acids.

#### Chromatography method development

**Fig. 4:**
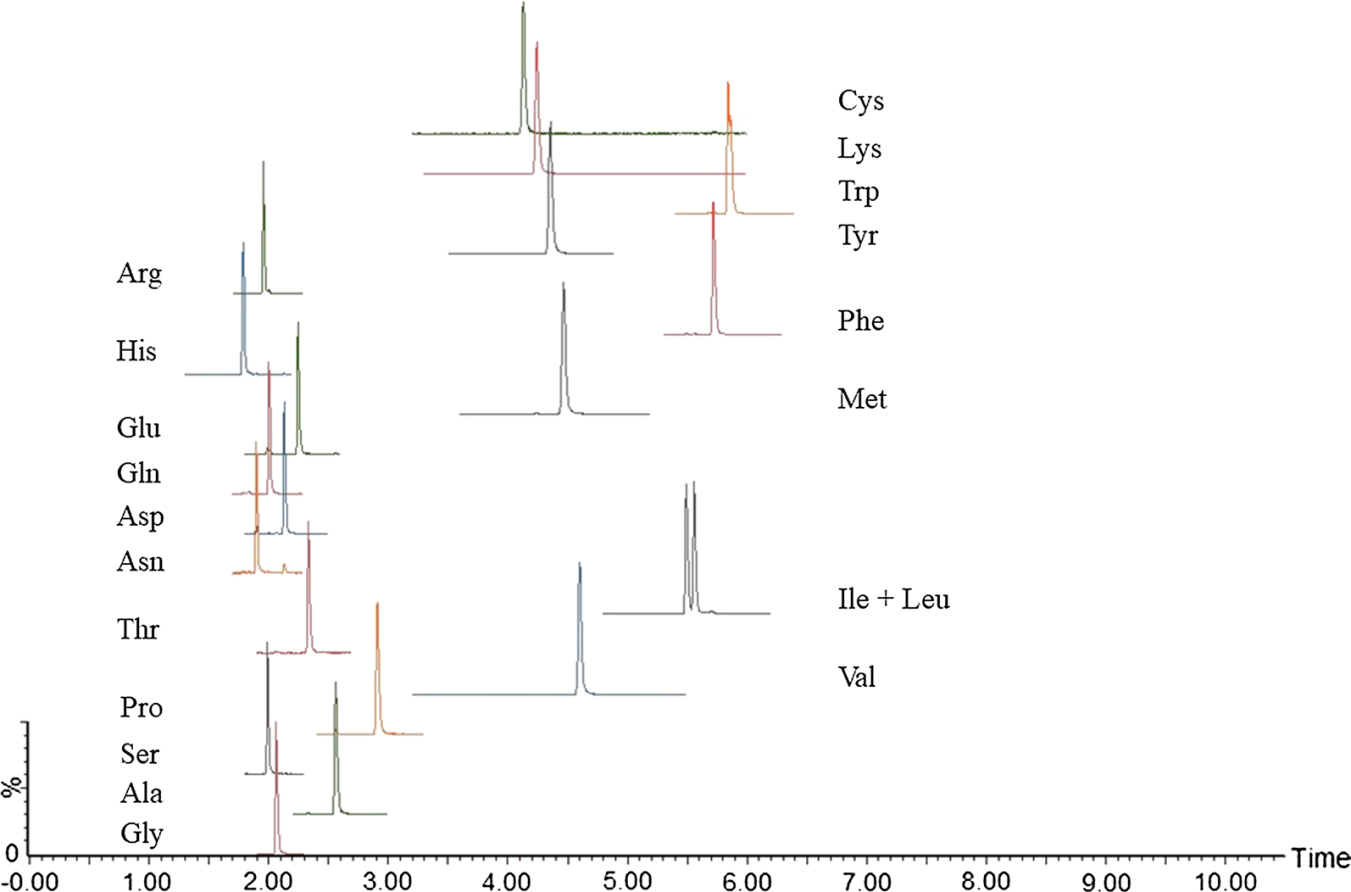
Chromatogram of the derivatized standards of 20 amino acids obtained with the use of UPLC-MS method. Concentration of the sample was 100 µM. For the chromatographic and detection conditions see “Instrumentation and analytical conditions”

A reversed-phase UHPLC method was developed to analyze derivatized amino acids products. Three different separation columns were tested in the method development process, namely Kinetex C18 (100 × 2.1 mm, 1.7 µm), Acquity UPLC HSS T3 (100 × 2.1 mm, 1.8 µm), and Cortecs UPLC C18 (100 × 2.1 mm, 1.6 µm). The ideal chromatographic conditions are characterized by the balance of retention of the derivatized amino acids products, time of analysis and appropriate resolution of analytes. Parameters such as retention time, efficacy and resolution between the isobaric amino acids—leucine and isoleucine—were therefore evaluated during the stationary phase optimization procedure. Results are summarized and compared in Table 2. According to the obtained results, the Cortecs UPLC C18 column was selected—the best retention and resolution parameters and the excellent peak shape of the amino acids derivative products were obtained. Individual peaks at separate mass channels were achieved, and isobaric amino acids isoleucine and leucine were separated with satisfactory resolution (R = 1.72) that is sufficient for quantification purposes. The use of a Cortecs C18 stationary phase was also accompanied with a short time of analysis—all analyzed compounds eluted in time lower than 6 min (the total run time was 10.5 min). This parameter is essential if there is a demand for high-throughput analysis. Illustrative chromatograms of derivative amino acids obtained under optimal separation conditions are presented in Fig. 4.

**Table 2:** Data obtained from the optimization procedure of the stacionary phase

	Kinetex C18				Acquity UPLC HSS T3				Cortecs UPLC C18			
	RT (min)	w_1/2_ (min)	N	Resolution Ile-Leu	RT (min)	w_1/2_ (min)	N	Resolution Ile-Leu	RT (min)	w_1/2_ (min)	N	Resolution Ile-Leu
Gly	1.97	0.03	23,889		2.26	0.03	31,440		2.06	0.0295	27,015	
Ala	2.27	0.03	31,719		2.68	0.03	44,212		2.56	0.0295	41,720	
Ser	1.91	0.03	22,456		2.17	0.04	16,305		1.99	0.02	54,847	
Pro	2.44	0.035	26,925		2.96	0.04	30,337		2.91	0.02	117,283	
Val	3.58	0.05	28,401		4.68	0.045	59,920		4.60	0.03	130,252	
Thr	2.13	0.03	27,927		2.45	0.025	53,206		2.33	0.025	48,122	
Cys	2.87	0.03	50,703		3.50	0.035	55,400		4.13	0.03	104,995	
Ile	4.97	0.045	67,577	1.49	5.71	0.04	112,892	1.52	5.49	0.03	185,529	1.72
Leu	5.09	0.05	57,412		5.80	0.03	207,073		5.57	0.025	275,005	
Asn	1.81	0.03	20,166		2.08	0.025	38,349		1.89	0.025	31,663	
Asp	2.00	0.035	18,090		2.28	0.025	46,079		2.13	0.025	40,215	
Gln	1.90	0.035	16,326		2.14	0.025	40,594		2.00	0.02	55,400	
Glu	2.08	0.03	26,631		2.35	0.03	33,994		2.24	0.02	69,494	
Lys	3.03	0.035	41,520		3.68	0.035	61,244		4.24	0.03	110,662	
Met	3.47	0.035	54,454		4.50	0.04	70,116		4.47	0.03	122,994	
His	1.71	0.025	25,919		1.98	0.025	34,750		1.79	0.02	44377	
Phe	5.30	0.05	62,247		5.97	0.025	315,921		5.72	0.03	201,400	
Arg	1.90	0.03	22,222		2.11	0.03	27,405		1.96	0.02	53,206	
Tyr	3.31	0.05	24,279		4.20	0.04	61,079		4.36	0.035	147,967	
Trp	5.50	0.06	46,551		6.11	0.03	229,800		5.84	0.025	302,312

Cortecs columns are packed with solid-core C18 reversed-phase particles with a mean particle diameter of 1.6 µm and have balanced retention of acids, bases, and neutrals at low and mid-range pH. These columns are characterized by increased efficiency, resolution and high throughput what was demonstrated in some recent papers that deal whit analysis of amino acids in plant samples [34], immunosuppressants in whole blood samples [40] or theoretical aspects of column efficiency [41]. According to these findings and parameters which are supported by our experiments, such stationary phase represents an appropriate approach for analysis of derivative amino acids.

### Method validation

The developed UHPLC–MS method was validated according to the ICH Q2(R1) guideline [42], and the obtained parameters from the validation procedure are summarized in Tables 3, 4 and 5. Calibrations were performed using six standards, and the linear calibration equation (y = ax + b) was generated using the ratio of analyte peak area over internal standard (IS) peak area after quantitative integration by TargetLynx XS software. The calibration lines showed excellent linearity over the studied concentration range what is demonstrated by the values of the coefficients of determination (r^2^) ranging in the interval of 0.9918–0.9998.

**Table 3:** Selected operation and validation parameters of the developed UHPLC-MS method for analysis of amino acids

	Mass (m/z)	t_R_ (min)	Range (µM)	Calibration line	Linearity (r^2^)	LOD (µM)	LOQ (µM)
Glycine	246.1	2.06	1–500	y = 0.0190x + 1.0335	0.9995	0.009	1
Alanine	260.1	2.56	1–500	y = 0.0196x + 0.0057	0.9998	0.185	1
Serine	276.1	1.99	1–500	y = 0.0205x − 0.0088	0.9993	0.263	1
Proline	286.1	2.91	1–500	y = 0.0193x − 0.0140	0.9998	0.551	1
Valine	288.1	4.60	1–500	y = 0.0197x + 0.0151	0.9998	0.168	1
Threonine	290.1	2.33	1–500	y = 0.0202x + 0.0237	0.9991	0.287	1
Cysteine	581.0	4.13	2.5–250	y = 0.0226x − 0.1034	0.9979	0.822	2.5
Isoleucine	302.1	5.49	1–500	y = 0.0214x + 0.0333	0.9998	0.218	1
Leucine	302.1	5.56	1–500	y = 0.0222x − 0.0107	0.9997	0.205	1
Asparagine	303.1	1.90	1–500	y = 0.0951x + 1.3291	0.9918	0.656	1
Aspartic acid	304.1	2.13	1–500	y = 0.0197x + 0.1095	0.9990	0.532	1
Glutamine	317.1	2.01	1–500	y = 0.0152x + 0.0115	0.9992	0.735	1
Lysine	487.1	4.25	1–500	y = 0.0147x − 0.0021	0.9994	0.368	1
Glutamic acid	318.1	2.24	1–500	y = 0.0210x − 0.0184	0.9996	0.204	1
Methionine	320.1	4.47	1–500	y = 0.0229x + 0.0056	0.9998	0.331	1
Histidine	326.1	1.79	1–500	y = 0.0141x − 0.0121	0.9995	0.127	1
Phenylalanine	336.1	5.72	1–500	y = 0.0166x − 0.0287	0.9995	0.153	1
Arginine	345.1	1.96	1–500	y = 0.0208x − 0.0114	0.9990	0.291	1
Tyrosine	352.1	4.36	1–500	y = 0.0161x − 0.0157	0.9998	0.128	1
Tryptophan	375.1	5.86	1–500	y = 0.0223x + 0.0663	0.9997	0.108	1

LOD, limit of detection; LOQ, limit of quantification; t_R_, retention time

**Table 4:** Accuracy and precision parameters of the developed UHPLC–MS method

	Within-run, n = 6			Between-run, n = 12		
	Mean found concentration (µM)	RE (% Nom.)	RSD (%)	Mean found concentration (µM)	RE (% Nom.)	RSD (%)
Glycine						
5 µM	4.50	− 9.00	3.14	4.90	− 2.00	9.72
50 µM	50.45	0.90	7.99	49.07	− 1.86	5.59
250 µM	254.55	1.82	6.81	240.60	− 3.76	6.69
Alanine						
5 µM	4.70	− 6.00	3.01	4.55	− 9.00	8.98
50 µM	49.25	− 1.50	0.43	48.87	− 2.26	1.70
250 µM	256.30	2.52	2.59	247.52	− 0.99	3.05
Serine						
5 µM	4.55	− 9.00	7.77	4.68	− 6.40	6.39
50 µM	49.15	− 1.70	2.45	48.45	− 3.10	4.04
250 µM	253.50	1.40	2.79	253.1	1.24	2.77
Proline						
5 µM	4.80	− 4.00	2.95	4.78	− 4.40	2.78
50 µM	49.90	− 0.20	7.09	50.63	1.26	3.85
250 µM	259.75	3.90	2.37	252.35	0.94	3.77
Valine						
5 µM	4.78	− 4.4	5.51	4.73	− 5.40	4.56
50 µM	48.90	− 2.20	2.60	50.65	1.30	4.12
250 µM	249.85	− 0.06	0.25	245.90	− 1.64	2.00
Threonine						
5 µM	4.95	− 1.00	7.14	4.62	− 7.60	7.43
50 µM	49.95	− 0.10	0.71	49.05	− 1.90	3.23
250 µM	251.25	0.50	1.21	240.7	− 3.72	3.77
Cysteine						
2.5 µM	2.60	4.00	5.44	2.53	1.20	4.11
25 µM	25.05	0.20	3.11	24.73	− 1.08	4.22
125 µM	122.78	− 1.78	2.85	120.44	− 3.65	2.45
Isoleucine						
5 µM	5.05	1.00	1.40	4.98	− 0.40	3.90
50 µM	49.60	− 0.80	3.42	50.45	0.90	2.73
250 µM	258.35	3.34	4.79	247.37	− 1.05	4.24
Leucine						
5 µM	4.85	− 3.00	1.46	4.72	− 5.60	4.91
50 µM	48.95	− 2.10	3.61	48.92	− 2.16	1.67
250 µM	252.95	1.18	5.40	243.43	− 2.63	4.32
Asparagine						
5 µM	5.30	6.00	8.22	4.98	− 0.40	11.57
50 µM	53.75	7.50	0.13	52.60	5.20	1.93
250 µM	249.75	− 0.10	2.58	242.53	− 2.99	2.64
Aspartic acid						
5 µM	4.65	− 7.00	1.52	4.43	− 11.40	11.31
50 µM	51.35	2.70	4.82	47.82	− 4.36	6.73
250 µM	262.40	4.96	11.97	247.03	− 1.19	7.91
Glutamine						
5 µM	4.85	− 3.00	1.46	4.53	− 9.40	5.52
50 µM	53.25	6.50	2.26	52.48	4.96	2.20
250 µM	245.15	− 1.94	5.51	245.32	− 1.87	3.18
Lysine						
5 µM	4.95	− 1.00	6.70	4.85	− 3.00	6.22
50 µM	52.00	4.00	1.90	51.62	3.24	3.29
250 µM	252.70	1.08	0.39	246.60	− 1.36	3.71
Glutamic acid						
5 µM	4.75	− 5.00	1.49	4.67	− 6.60	1.75
50 µM	50.20	0.40	8.48	48.28	− 3.44	4.07
250 µM	261.10	4.44	6.07	260.27	4.11	3.15
Methionine						
5 µM	4.75	− 5.00	1.49	4.97	− 0.60	4.35
50 µM	50.20	0.40	5.92	49.03	− 1.94	4.25
250 µM	253.20	1.28	6.26	245.90	− 1.64	3.88
Histidine						
5 µM	4.63	− 7.40	10.65	4.72	− 5.60	9.40
50 µM	50.60	1.12	2.80	49.05	− 1.90	4.71
250 µM	251.70	0.68	2.59	249.07	− 0.37	2.22
Phenylalanine						
5 µM	4.85	− 3.00	1.46	4.80	− 4.00	1.86
50 µM	47.85	− 4.30	4.29	46.45	− 7.10	3.56
250 µM	258.00	3.20	1.21	249.48	− 0.21	3.79
Arginine						
5 µM	4.95	− 1.00	4.29	4.62	− 7.60	7.17
50 µM	48.45	− 3.10	2.19	48.77	− 2.46	6.86
250 µM	258.15	3.26	0.36	245.08	− 1.97	5.06
Tyrosine						
5 µM	4.77	− 4.60	1.21	4.63	− 7.40	3.53
50 µM	49.30	− 1.40	3.44	47.22	− 5.56	4.11
250 µM	256.05	2.42	6.43	246.23	− 1.51	5.26
Tryptophan						
5 µM	5.15	3.00	1.37	4.98	− 0.40	6.14
50 µM	49.55	− 0.90	2.14	50.40	0.80	2.70
250 µM	263.90	5.56	9.59	254.52	1.81	5.57

**Table 5:** Evaluation of samples at 3 concentration levels for estimation of stability and recovery of the UHPLC–MS method

	Freeze–thaw stability (3 cycles)		Autosampler stability (48 h)		Recovery	
	Concentration found (µM)	Accuracy (%RE)	Concentration found (µM)	Accuracy (%RE)	Found concentration (µM)	Recovery (%)
Glycine						
5 µM	5.20	4.00	5.25	5.00	4.70	94.0
70 µM	69.15	− 1.21	72.80	4.00	64.15	91.6
300 µM	302.70	0.90	299.05	− 0.30	288.40	96.1
Alanine						
5 µM	5.35	7.00	4.90	− 2.00	4.70	94.0
70 µM	71.35	1.93	66.80	− 4.57	70.55	100.8
300 µM	300.65	0.22	299.15	− 0.28	296.75	98.9
Serine						
5 µM	5.10	2.00	4.85	− 3.00	4.60	92.0
70 µM	65.55	− 6.36	69.05	− 1.36	68.00	97.1
300 µM	297.05	− 0.98	304.80	1.60	298.10	99.4
Proline						
5 µM	5.10	2.00	4.90	− 2.00	4.70	94.0
70 µM	71.15	1.64	70.30	− 0.43	69.60	99.4
300 µM	303.90	1.30	293.00	− 2.33	296.80	98.9
Valine						
5 µM	5.20	4.00	5.00	0.00	5.05	101.0
70 µM	70.70	1.00	67.35	− 3.79	68.75	98.2
300 µM	304.45	1.48	300.65	0.22	295.65	98.6
Threonine						
5 µM	4.95	− 1.00	4.90	− 2.00	4.95	99.0
70 µM	68.35	− 2.36	67.95	− 2.93	67.45	96.36
300 µM	305.75	1.92	301.30	0.43	299.85	99.9
Cysteine						
2.5 µM	2.43	− 2.80	2.43	− 2.80	2.40	96.0
35 µM	34.23	− 2.20	33.15	− 5.29	33.98	97.1
150 µM	148.50	− 1.00	148.70	− 0.87	141.73	94.5
Isoleucine						
5 µM	5.05	1.00	4.95	− 1.00	5.00	100.0
70 µM	71.40	2.00	70.65	0.93	71.20	101.7
300 µM	302.45	0.82	305.40	1.80	302.20	100.7
Leucine						
5 µM	5.10	2.00	4.95	− 1.00	5.00	100.0
70 µM	70.95	1.36	70.55	0.79	71.95	102.8
300 µM	305.85	1.95	299.10	− 0.30	302.30	100.8
Asparagine						
5 µM	4.95	− 1.00	4.80	− 4.00	4.70	94.0
70 µM	71.95	2.79	68.10	− 2.71	67.60	96.6
300 µM	287.85	− 4.05	296.55	− 1.15	292.20	97.4
Aspartic acid						
5 µM	5.30	6.00	4.85	− 3.00	4.85	97.0
70 µM	67.80	− 3.14	67.45	− 3.64	63.40	90.6
300 µM	305.3	1.77	307.70	2.57	292.15	97.4
Glutamine						
5 µM	4.75	− 5.00	4.95	− 1.00	4.65	93.0
70 µM	71.15	1.64	68.15	− 2.64	67.35	96.2
300 µM	295.15	− 1.62	297.85	− 0.72	287.85	96.0
Lysine						
5 µM	5.0	0.00	4.85	− 3.00	4.75	95.0
70 µM	69.25	− 1.07	67.20	− 4.00	68.15	97.4
300 µM	309.45	3.15	287.35	− 4.22	285.20	95.1
Glutamic acid						
5 µM	4.75	− 5.00	4.90	− 2.00	4.80	96.0
70 µM	65.50	− 6.43	68.10	− 2.71	70.20	100.3
300 µM	298.35	− 0.55	300.70	0.23	294.35	98.1
Methionine						
5 µM	4.75	− 5.00	5.00	0.00	4.95	99.0
70 µM	69.70	− 0.43	67.45	− 3.64	66.30	94.7
300 µM	294.90	− 1.70	299.50	− 0.17	295.20	98.4
Histidine						
5 µM	4.40	− 12.00	4.85	− 3.00	4.50	90.0
70 µM	71.70	2.43	67.10	− 4.14	64.65	92.4
300 µM	297.20	− 0.93	303.90	1.30	295.70	98.6
Phenylalanine						
5 µM	4.90	− 2.00	4.80	− 4.00	4.85	97.0
70 µM	69.95	− 0.07	67.45	− 3.64	65.55	93.6
300 µM	306.10	2.03	304.95	1.65	292.75	97.6
Arginine						
5 µM	5.0	0.00	4.90	− 2.00	5.00	100.0
70 µM	70.05	0.07	69.25	− 1.07	69.15	98.8
300 µM	297.55	− 0.82	301.70	0.57	294.95	98.3
Tyrosine						
5 µM	4.85	− 3.00	4.85	− 3.00	5.00	100.0
70 µM	70.15	0.21	66.70	− 4.71	67.70	96.7
300 µM	299.95	− 0.02	306.50	2.17	297.70	99.2
Tryptophan						
5 µM	5.10	2.00	5.05	1.00	5.05	101.0
70 µM	70.80	1.14	67.80	− 3.14	71.35	101.9
300 µM	298.50	− 0.50	297.80	− 0.73	294.85	98.3

Limits of detection (LOD) and limits of quantification (LOQ) were calculated from standard chromatograms as the signal to noise (S/N) ratios having values 3 and 10, respectively. The obtained LOD values were in the range of 0.009–0.822 µM and LOQ values were in the range of 1–2.5 µM (Table 3). These values were more than sufficient for the analysis of amino acids in standard proteins and commercially available biopharmaceutical samples. The LOQ value of amino acid Cys was higher in comparison to the LOQ values of other determined amino acids. This fact can be associated with the chemical nature of this amino acid and its responsible properties. Specifically, the thiol group is unstable and makes Cys a highly reactive compound which is easily transformed into mixed and symmetrical disulfides.

The precision of the developed method was determined as repeatability (intra-assay precision) and intermediate precision. Repeatability was evaluated according to the analysis of six repeated injections of three different concentrations of the analytes. Intermediate precision was determined by analysis of three different concentrations over 6 days. The calculated RSD values obtained from the intraday and interday precision assay were in the range of 0.25–11.97% and 1.67–11.57%, respectively and can be found in detail in Table 4. The intraday precision represents the precision under the same operating conditions over a short interval of time—in 1 day. On the other side, the interday precision expresses precision within-laboratory variations—e.g., different days. Therefore, the results obtained from this validation procedure may be different and are very often affected by the operation conditions—preparation of the samples by other analysts, the performance of the experiments in different days and by another analyst.

The accuracy was evaluated with the recovery test. Individual samples of Waters hydrolyzed standard were spiked with the mixture of amino acids standards at three different concentration levels, and the recoveries were calculated based on the difference between the amount determined in the spiked samples and the amount observed in the non-spiked samples. The obtained results were in the range 90–102.8% which demonstrates excellent recovery of the developed UHPLC–MS approach and negligible influence of the pharmaceutical matrix on the analytical signal of amino acids. The results of stability studies also indicate that derivatized AAs were stable at least for 48 h when stored at 10 °C in autosampler and during the three freeze/thaw cycles at − 20 °C (Table 5).

### Method application

#### Amino acid standard, hydrolysate

Amino acid hydrolysate standard is a commercially available mixture of 17 hydrolyzed amino acids at 2.5 mM concentration level except for cysteine which is present at 1.25 mM concentration level. The results obtained from the analysis of 10-times dilute sample are summarized in Table 6. The determined content of each amino acid present in the sample ranged from 91 to 104%.

**Table 6:** Quantification of amino acids present in model sample of Waters amino acid standard hydrolysate

	Expected concentration [µM]	AA standard hydrolysate [µM]	Recovery [%]
Glycine	250	249.35 ± 3.04	99.7
Alanine	250	255.85 ± 4.17	102.3
Serine	250	245.45 ± 3.18	98.2
Proline	250	256.85 ± 3.47	102.7
Valine	250	237.25 ± 3.47	94.9
Threonine	250	251.25 ± 4.31	100.5
Cysteine	125	125.05 ± 3.89	100.0
Isoleucine	250	255.10 ± 4.10	102.0
Leucine	250	244.45 ± 1.49	97.8
Aspartic acid	250	239.75 ± 1.34	95.9
Lysine	250	245.60 ± 2.12	98.2
Glutamic acid	250	227.57 ± 10.43	91.0
Methionine	250	247.90 ± 3.96	99.2
Histidine	250	260.60 ± 1.27	104.2
Phenylalanine	250	237.75 ± 0.64	95.1
Arginine	250	246.00 ± 2.12	98.4
Tyrosine	250	236.5 ± 3.54	94.6

#### Model protein sample—Bovine serum albumin

**Fig. 5:**
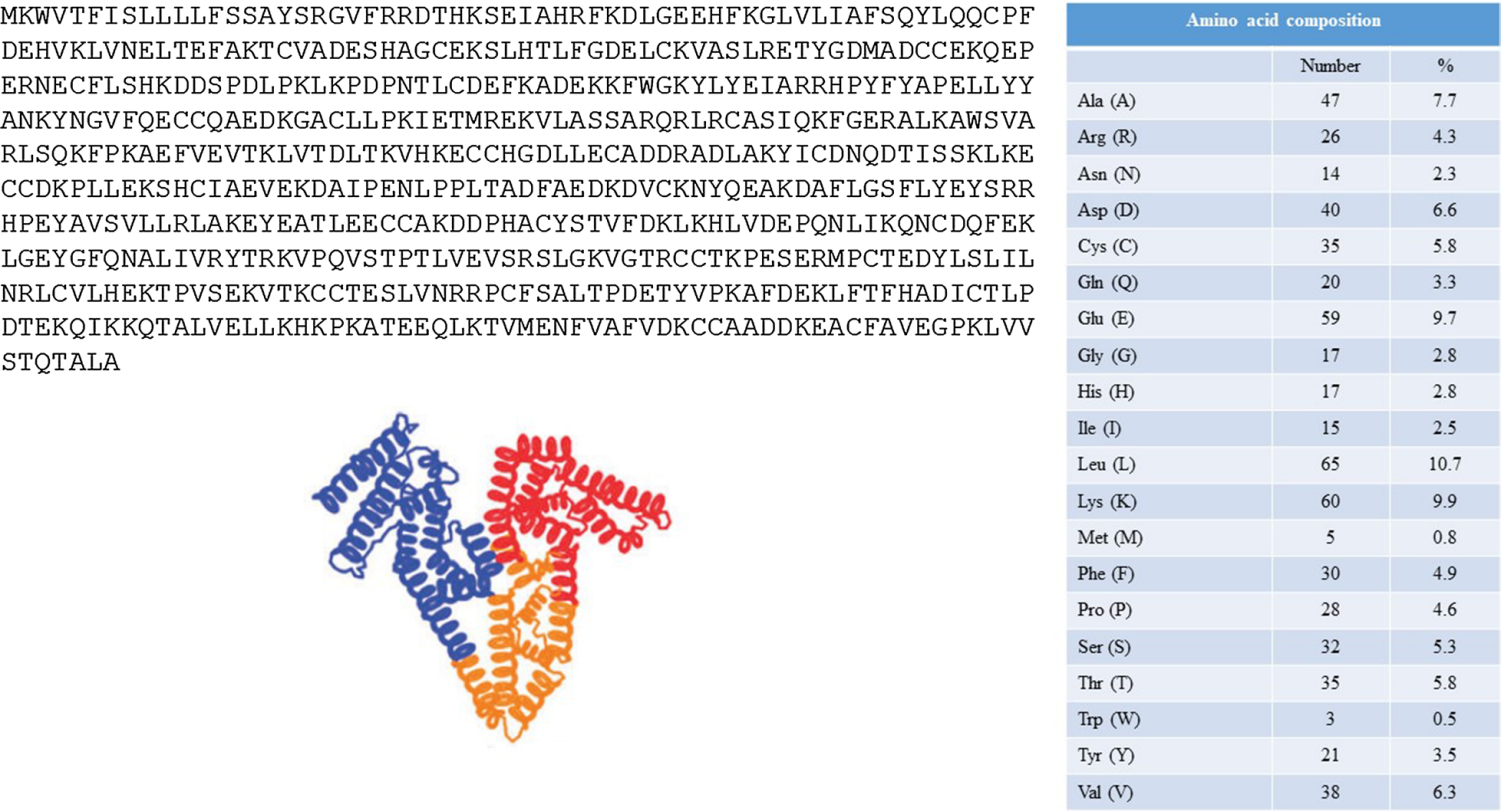
Illustrative scheme of the bovine serum albumin—BSA (adapted from [39]) with the corresponding amino acids sequence and abundance of each amino acid

The developed and validated UHPLC–MS method for fast and reliable quantification of amino acids was tested on hydrolyzed model protein sample which was represented by bovine serum albumin (BSA) sample. BSA is commonly very often used in different protein assay protocols such as BCA or Bradford protein assay. Furthermore, BSA is also used as a model protein because of its stability, its lack of effect in many biochemical reactions and its low cost [43]. The AA sequence and the illustrative structure of BSA are in detail depicted in Fig. 5. In our experiments, we determined the concentration of model BSA sample prepared at 5 mg mL^−1^ concentration level by simple dilution of a known amount of BSA standard in LC–MS grade water. The determined concentration by the developed AAA method was 4.83 ± 0.43 mg mL^−1^ (Table 7).

**Table 7:** Quantification of different protein and biopharmaceutical samples with the use of well recovered amino acids

	BSA	KLH batch no. 1	KLH batch no. 2	KLH batch no. 3	Enbrel
Alanine	4.88	19.85	20.36	20.76	48.74
Leucine	4.86	19.00	21.09	20.57	45.85
Asparagine	4.32	19.84	21.35	19.27	49.77
Aspartic acid					
Glutamine	5.25	20.09	19.98	20.13	49.45
Glutamic acid					
Phenylalanine	4.33	21.37	18.92	19.93	53.74
Arginine	4.71	20.26	18.27	19.55	–
Tyrosine	5.48	20.37	20.09	19.71	49.54
Determined concentration	4.83 ± 0.43 mg mL^−1^	20.11 ± 0.71 mg mL^−1^	20.01 ± 1.10 mg mL^−1^	19.99 ± 0.54 mg mL^−1^	49.52 ± 2.53 mg mL^−1^
Expected concentration	5 mg mL^−1^	20 mg mL^−1^	20 mg mL^−1^	20 mg mL^−1^	50 mg mL^−1^

#### Keyhole limpet hemocyanin—preparation Vacmune^®^

**Fig. 6:**
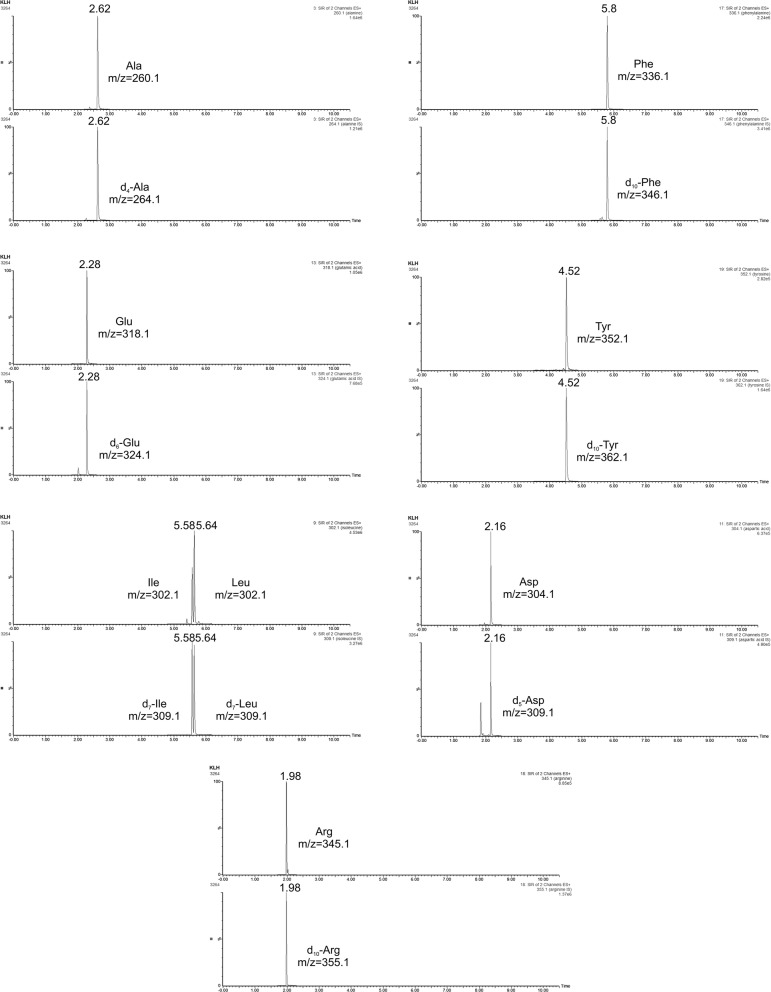
Selected ion chromatograms of 7 well recovered amino acids and their isotope labelled internal standards in KLH hydrolyzed sample—preparation Vacmune^®^. For the chromatographic and detection conditions see “Instrumentation and analytical conditions”

In our work, we determined the concentration of KLH in three commercially available samples (preparation Vacmune^®^) from the company biosyn Arzneimittel GmbH. The determination of protein content in KLH samples was performed with the use of well-recovered amino acids—Asx, Glx, Arg, Ala, Tyr, Leu, and Phe. The determined concentration of three different KLH samples was 20.11 ± 0.71 mg mL^−1^, 20.01 ± 1.10 mg mL^−1^ and 19.99 ± 0.54 mg mL^−1^, respectively (Table 7). This is in a good agreement with the concentration of the protein declared by the manufacturer (20 mg mL^−1^). Illustrative selected ion chromatograms of the well recovered amino acids and their internal standards obtained during the analysis of KLH sample are in Fig. 6.

#### Biopharmaceutical sample—Enbrel

According to the previous encouraging results from the AAA of the model samples of hydrolyzed amino acids standards and hydrolyzed bovine serum albumin, the developed UHPLC–MS method was also applied for protein quantification of the biopharmaceutical Enbrel.

The manufacturer declares the content of etanercept at 50 mg mL^−1^ concentration level. The biopharmaceutical Enbrel is formulated with content of l-arginine at a specific concentration that acts as a stabilizing agent. Therefore, higher concentration for this amino acid was expected and this amino acid was not used for calculation of the etanercept concentration in the analyzed samples. The determined concentration of etanercept with our method was 49.52 ± 2.53 mg mL^−1^ (Table 7).

## Concluding remarks

A simple and rapid approach which combines fast precolumn derivatization with RP-UHPLC–MS analysis was presented as an effective and powerful tool for quantitative amino acid analysis in different protein and biopharmaceutical samples. The use of AccQTag Ultra derivatization reagent leads to improved separation and detection properties of the analytes. Combination of UHPLC and simple QDa mass spectrometry detector enables very fast analysis. Moreover, the use of stable isotope labeled internal standards improves the reproducibility and reliability of the method. These statements were confirmed by the results obtained from the analysis of three different protein samples—model sample of BSA, immunogen KLH, and biopharmaceutical etanercept. Thanks to the simplicity of the analytical procedure, short analysis time, minimum sample consumption, high sensitivity, low obtained limits of detection and no requirement on highly trained personnel, the presented approach represent a cheaper alternative to the recently used AAA based on LC–MS combination with the use of more advanced mass spectrometers. These attributes indicate the possibility for implementation of the developed UHPLC–MS as a routine method for the quality control laboratories, and after some modification, there is a good possibility for its adaptation into the field of clinical and biomedical analysis.

## Data Availability

All data including the original mass spectrometry files are fully available upon request.

## Abbreviations

5-AIQC: 5-aminoisoquinolyl-*N*-hydroxysuccinimide hydrochloride
AA: amino acid
AAA: amino acid analysis
AQC: 6-aminoquinolyl-*N*-hydroxysuccinimide hydrochloride
BSA: bovine serum albumin
d_3_-DMABS: dimethyl-1,1,1-d_3_-aminobutyryl succinimide hydrochloride
d_6_-DMABS: dimethyl-d_6_-aminobutyryl succinimide hydrochloride
Dansyl-Cl: 5-(dimethylamino)-naphthalene-1-sulfonyl chloride
ESI: electrospray ionization
FDA: U.S. Food and Drug Administration
FMOC-Cl: 9-fluorenylmethyl chloroformate
HIV: human immunodeficiency virus
ICH: International Council for Harmonisation
KLH: keyhole limpet hemocyanin
LC: liquid chromatography
LOD: limit of detection
LOQ: limit of quantification
MS: mass spectrometry/mass spectrometer
OPA: *O*-phthaladehyd
PITC: phenylisothiocyanate
RP: reversed-phase
SIM: single ion-monitoring
TNFR: tumor necrosis factor receptor
UHPLC: ultra high-performance liquid chromatography
